# Risk factor analysis of device-related infections: value of re-sampling method on the real-world imbalanced dataset

**DOI:** 10.1186/s12911-019-0899-4

**Published:** 2019-09-11

**Authors:** Xiang-Fei Feng, Ling-Chao Yang, Li-Zhuang Tan, Yi-Gang Li

**Affiliations:** 1 Department of Cardiology, Xinhua Hospital, School of Medicine, Shanghai Jiao Tong University, 1665#, KongJiang Road, Shanghai, 200092 China; 2 School of Electronics and Information Engineering, Beijing Jiaotong University, Beijing, China

**Keywords:** Device, Infection, Risk factors, Implantation, Re-sampling technique

## Abstract

**Background:**

The incidence of cardiac implantable electronic device infection (CIEDI) is low and usually belongs to the typical imbalanced dataset. We sought to describe our experience on the management of the imbalanced CIEDI dataset.

**Methods:**

Database from two centers of patients undergoing device implantation from 2001 to 2016 were reviewed retrospectively. Re-sampling technique was used to improve the classifier accuracy.

**Results:**

CIEDI was identified in 28 out of 4959 procedures (0.56%); a high imbalance existed in the sizes of the patient profiles. In univariate analyses, replacement procedure and male were significantly associated with an increase in CIEDI: (53.6% vs. 23.4, 0.8% vs. 0.3%, *P* < 0.01). Multivariate logistic regression analysis showed that gender (odds ratio, OR = 3.503), age (OR = 1.032), replacement procedure (OR = 3.503), and use of antibiotics (OR = 0.250) remained as independent predictors of CIEDI (all *P* < 0.05) after adjustment for diabetes, post-operation fever, and device style, device company.

There were 616 under-sampled cases and 123 over-sampled cases in the analyzed cohort after re-sampling. The re-sampling and bootstrap results were robust and largely like the analysis results prior re-sampling method, while use of antibiotics lost the predicting capacity for CIEDI after re-sampling technique (*P* > 0.05).

**Conclusion:**

The application of re-sampling techniques can generate useful synthetic samples for the classification of imbalanced data and improve the accuracy of predicting efficacy of CIEDI. The peri-operative assessment should be intensified in male and aged patients as well as patients receiving replacement procedures for the risk of CIEDI.

## Background

Cardiac implantable electronic device infection (CIEDI) rates are increasing [1]. But the reported rates of CIEDI were still quite low, for 2017 which were ≤ 2% in 78.7% of the centers, while exceeded 5% only in 7.8% of the centers [1]. So, events, such as CIEDI, are not frequent, that means inherently rare or hard to collect (rare events) [2, 3]. Due to this, the observed data is usually severely unbalanced [4]. A database is imbalanced if the underlying clusters are not equally represented [5]. A common threshold to determine this scenario is when the ratio between the largest class and the smaller class is ≥ 1.5 [6]. In practical applications, the ratio of the small to the large can be 1 to 100, 1 to 1000, or even more sometimes, that is highly imbalanced [4]. All of the real-world data sets are naturally imbalanced to a certain degree [7].

Previous study have examined imbalanced data with binary response variables containing many more non-events (zeros) than events (ones), and showed that these variables are difficult to predict and explain [8]. The problem is that maximum likelihood estimation of the logistic model is well-known to suffer from small-sample bias. And the degree of bias is strongly dependent on the number of cases in the less frequent of the two categories. For most imbalanced datasets, the application of re-sampling method improves classifier accuracy [5]. Random oversampling and under-sampling are two of the most common re-sampling techniques [9].

In case of rare positive instances, appropriate over-sampling strategy seems to be effective and preferable. For more general class imbalance problems with sufficient instances of the minority class, under-sampling strategy is more widely used [10].

Accordingly, in this study, a re-sampling method was applied to determine CIEDI -associated risk factors from retrospective imbalanced databases of patients undergoing CIED implantation from 2001 to 2016 in two medical centers.

## Methods

### Data source

The Queen Mary Hospital and the Xinhua Hospital are the ones of the biggest hospitals in Hong Kong and Shanghai, respectively. A retrospective database review was conducted in CIED clinics on all the patients who had CIED implantation at the hospitals from January 1, 2001 to June 30, 2016. The study was approved by Ethics Committee of Xinhua Hospital Affiliated to Shanghai Jiaotong University School of Medicine (approval number: XHEC-D-2017-056) and performed in accordance with the Declaration of Helsinki.

Patients with permanent single-chamber or dual-chamber pacemakers, implantable cardioverter-defibrillator (ICD) or cardiac resynchronization therapy (CRT) device were included in this study.

### Study design

**Fig. 1:**
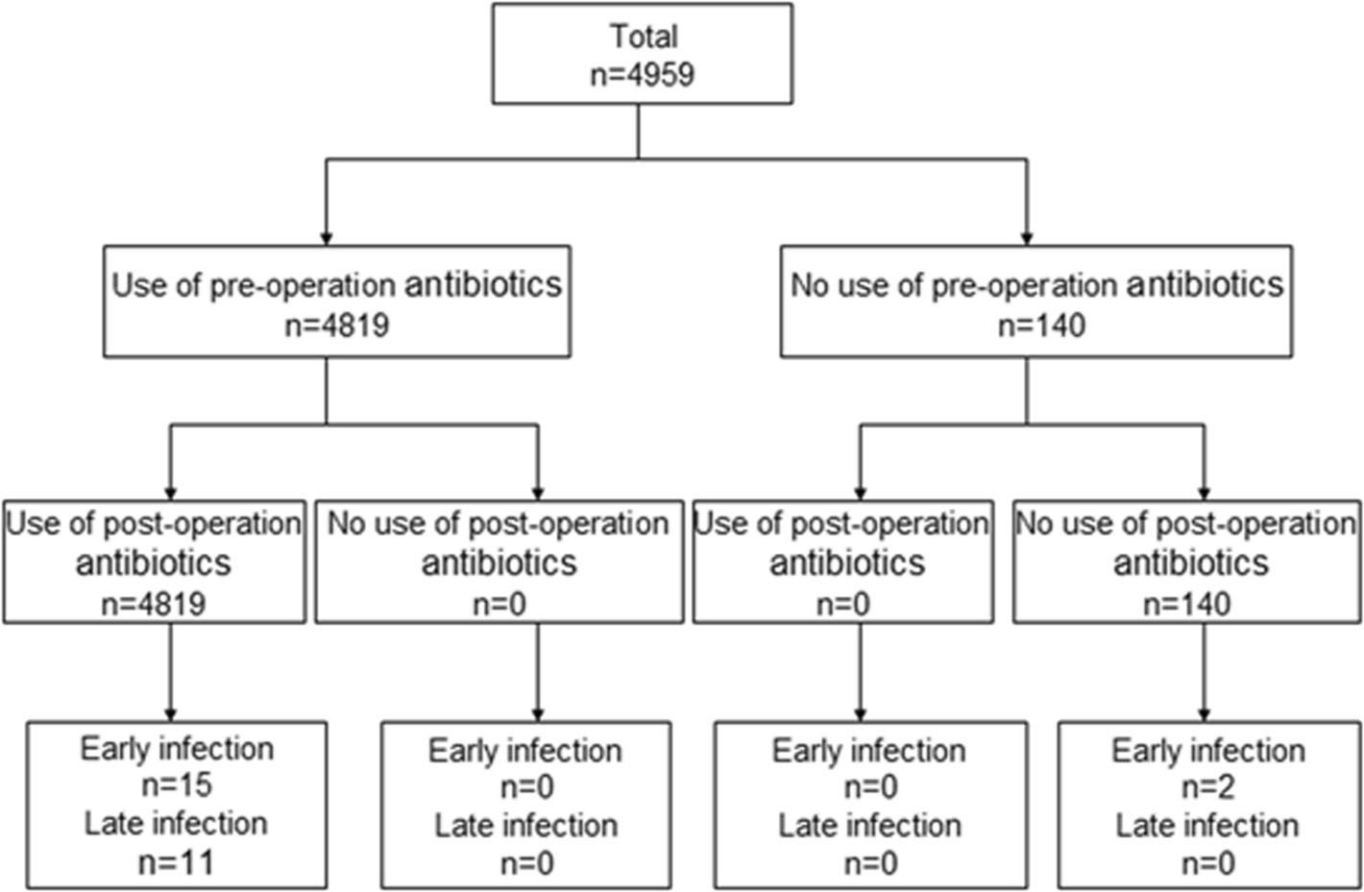
Study design and flow chart

Study design was showed in Fig. 1. In the analysis of antibiotics administration type, the patients were divided into three groups: prophylaxis antibiotics free, pre-procedure antibiotics and post-procedure antibiotics. The choice of antibiotics was based on the belief that skin flora would be the most likely contaminant and *S. aureus* is the most common microorganism to cause superficial infection [11]. The duration of antibiotic therapy was empirical choice and the recommended duration was pragmatic [12].

CIEDI was defined in the previous expert consensus with criteria [13]. Briefly, We selected patients with indications of localized (pacemaker site tenderness, swelling or erythema, skin erosion, or migration from the pocket) or systemic infection (bacteremia and/or endocarditis) [14]. Ultra-early infection was defined when CIEDI developed within one month after a new implantation, while early infection was defined within one year; and late infection was further defined when CIEDI developed more than one year after a new implantation [15].

### Follow-up

Data obtained during implantation and clinical follow-ups were entered into a database in the SPSS data management system and followed chronologically. The patients had long-term follow-ups at the CIED clinics. For the ones who could not be contacted, the dates of the last follow-ups were used as the corresponding period of follow-ups. For patients with multiple CIED surgeries, the most recent surgery was deemed as the procedure responsible for the infection.

### Statistical analysis

#### The primary analysis

The data were entered into a database formulated within the SPSS data management system (version 22.0, SPSS Inc., Chicago, Illinois). The continuous variables were expressed as the mean ± SD and were compared using a Student’s t-test. Comparisons between discrete variables were made using a χ^2^ test. Univariate analysis was performed for factors that may increase the rate of CIEDI, followed by a stepwise logistic regression including all univariate analysis with a *P*-value < 0.1. The association between study groups was examined using multivariable logistic regression analysis. All tests of significance were two sided, and P-value of < 0.05 was considered statistically significant. Receiver operating characteristic (ROC) curve analysis was also used to determine the optimal cut-off value of risk factors for the prediction of CIEDI.

#### Re-sampling analysis

In order to correct the skewness, we re-sampled the data by over-sampling and under-sampling according to the Synthetic Minority Over-sampling Technique (SMOTE) algorithm [9]. For the random under-sampling algorithm, the majority class was under-sampled by randomly removing 90% samples from the majority class. The random over-sampling method balances the ratio of the classes by copying instances from the minority class randomly. Then, these newly generated samples could be used in cluster-based approach to impute the missing observation in the original dataset for subsequent analysis [5]. ROCs were also evaluated by comparing the Area under Curve (AUC).

The bootstrap was a statistically elegant procedure that relies on random sampling with replacement. This technique allowed estimation of the sampling distribution of almost any statistic using random sampling methods [16]. In the end, the data was statistically analyzed by bootstrap method and the outcomes were estimated compared with direct measurements.

#### Accuracy rate testing

In this paper, three different classification algorithms of artificial neural network (ANN) [17], random forests (RF) [18], and support vector machines (SVM) [19] are used to verify the impact of re-sampling on accuracy rate. In order to ensure that there are enough training sets, and avoid losing the information of the original dataset, we do not adjust the number of majority class (non-CIEDI patients). By SMOTE algorithm, the number of minority class (CIEDI patients) is synthesized from minimum to approximate majority class (synthesized class). The majority class and the synthesized class are combined into a new database (re-sampling database). In order to reduce the impact of the number of dataset on the performance of the algorithm, we expanded the original dataset proportionally. For each database, we select 80% as a training set, 10% as a test set, and 10% as a validation set, and used F_1_-score value of validation set as the evaluation index, which is the harmonic mean of the accuracy rate and the recall rate, with a maximum of 1 and a minimum of 0. The larger the F_1_-score value, the more accurate the prediction of the model.

## Results

### Patient characteristics

During the study period, 5072 procedures were performed. Data from 113 patients were missing, and data from 4959 patients were analyzed (Mary 2273, Xinhua 2686). At the time of the procedures, the mean age of the patients was 72.5 ± 12.7 years. The gender distribution was as follows: male = 2628 (52.9%); female = 2331 (47.1%). CIEDI was identified in 28 (0.56%) patients. Time from device insertion to the initial symptoms and signs of infection were as follows: ultra-early infection = 6 (21.4%); early infection = 11 (39.3%); late infection = 11 (39.2%) (Fig. 1).

### The primary analysis

First, we dichotomized all patients according to the device infection or not (Table 1), and analyzed, then divided the CIEDI patients according to various clinical variables, such as gender, antibiotics, post-procedure fever and so on (Table 2) and analyzed.

**Table 1:** Clinical characteristics of device patients with infection or Un-infection group (*n* = 4959)

	Infection group (*n* = 28)	Un-infection group (*n* = 4931)	*P* value
Male, N (%)	22 (78.6%)	2606 (52.8%)	0.007
Female, N (%)	6 (21.4%)	2325 (47.2%)	0.007
Age (yrs), mean ± SD	65.5 ± 12.2	71.3 ± 14.2	0.178
Diabetes, n (%)	5 (17.9%)	951 (19.3%)	0.848
Pre-operation antibiotics, n (%)	26 (92.9%)	4754 (96.4%)	0.619
Post-operation antibiotics, n (%)	26 (92.9%)	4754 (96.4%)	0.619
Replacement procedure, n(%)	15 (53.6%)	1136 (23.0%)	0.001
Post-operation fever, n(%)	4 (14.3%)	414 (8.4%)	0.437
ICD, n(%)	2 (7.1%)	644 (13.1%)	0.518

*ICD* implantable cardioverter defibrillator

**Table 2:** Dichotomized device infection patients across clinical variables (*n*=28)

Clinical variables (Group I & Group II )	Group I	Group II	*P* value
	n (total,%)	n (total,%)	
Gender (male & female)	22 (2628,0.8)	6 (2331, 0.3)	0.007
Post-operation fever (yes & no)	4 (418,1.0)	24 (4541,0.5)	0.437
Antibiotics (yes & no)	26 (4819,0.5)	2 (140,1.4)	0.417
Device type (pacemaker & ICD)	26 (4313,0.6)	2 (646,0.3)	0.518
Type of procedure (primary & replacement)	13 (3705,0.4)	15 (1254,1.2)	0.001

*ICD* Implantable cardioverter defibrillator

From Table 1, we could see that among 28 CIEDI patients, 22 patients were male, 6 patients were female, and the mean age of the CIEDI patients was 65.5 ± 12.2 years, 15 patients were replacement procedure. Table 1 showed that the ratio of male and prevalence of replacement procedure were significantly higher in infection group than in un-infection group (all, *P* < 0.05). On the contrary, the following were not statistically different between infection group and un-infection group, such as age, diabetes, the use of antibiotics, and post-operation fever (all, *P* > 0.05).

From Table 2, we could see that among 28 infection cases, the infection rate in male was higher than in female (22/2628, 0.8% vs. 6/2331, 0.3%, *P* = 0.007), and the infection rate of replacement was higher than primary (15/1254, 1.2% vs. 13/3705, 0.4%, *P* = 0.001). While the followings were not statistically different between dichotomized groups, such as antibiotics, device type and post-operation fever (all P > 0.05). So, male gender and replacement procedure were significantly associated with CIEDI.

**Fig. 2:**
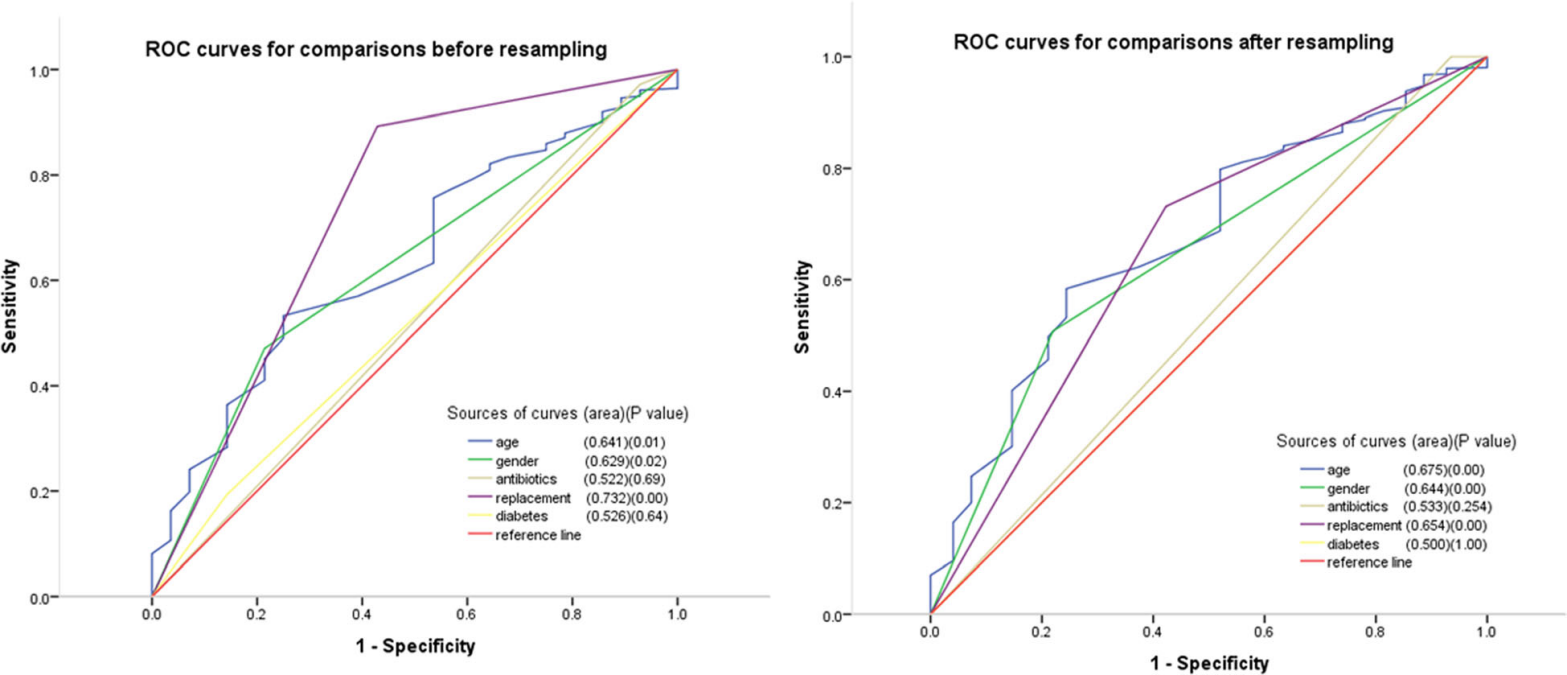
Comparison in ROC curves and AUCs between before (left) and after (right) re-sampling in prediction of CIEDI with similar variables. As shown in Figure, whether before or after re-sampling, replacement procedure, gender, and age all had large AUC. This suggests potential roles of them in prediction of patients at risks for CIEDI

In bivariate correlation analysis, the following factors were significantly associated with infection: gender (Correlation coefficient: 0.041) and age (0.036), replacement procedure (− 0.109), antibiotics (− 0.026). In multivariate logistic regression analysis controlling for postoperative fever at revision, gender (OR = 3.503, 95%CI: 1.712–7.169), age (OR = 1.032, 95% CI: 1.010–1.054), and replacement procedure (OR = 0.065, 95%CI: 0.032–0.132), and antibiotics (OR = 0.250, 95%CI: 0.72–0.863) also remained significant predictors of infection (*P* < 0.05) (Table 3). As shown in Fig. 2, a ROC curve analysis demonstrated that replacement procedure had an AUC = 73.2%, while gender and age had an AUC of less than 70%. This suggested a potential role of the replacement procedure in prediction of patients at risk for CIEDI.

**Table 3:** Multivariable logistic regression analysis before re-sampling (*n* = 4959)

Variables	Β	SE	OR	95%CI	*P* value
Age	0.031	0.011	1.032	1.010–1.054	0.004
gender	1.254	0.365	3.503	1.712–7.169	0.001
replacement procedure	−2.735	0.361	0.065	0.032--0.132	0.000
antibiotics	−1.386	0.632	0.250	0.72–0.863	0.028

*CI* confidence intervals, *SE* standard error

### The re-sampling analysis

Our data showed that there were 4959 device procedures with CIEDI identified in 28 (0.56%) patients, a high imbalance in the sizes of the patient profiles. According to the SMOTE algorithm, the majority class was under-sampled to 616 cases, and by copying instances, the minority class was over-sampled to 123 cases randomly. Then, these newly generated samples (*n* = 739) could be analyzed (Table 4).

**Table 4:** Clinical characteristics of device patients after re-sampling (*n* = 739)

	Infection group (*n* = 123)	Un-infection group (*n* = 616)	*P* value
Male, N (%)	96 (78.0%)	303 (49.2%)	0.001
Female, N (%)	27 (22.0%)	313 (50.8%)	0.001
Age (yrs), mean ± SD	67.3 ± 11.4	73.9 ± 11.3	0.001
Diabetes, n (%)	18 (16.7%)	90 (19.3%)	0.995
Pre-operation antibiotics, n (%)	115 (93.5%)	616 (100%)	0.001
Post-operation antibiotics, n (%)	115 (93.5%)	616 (100%)	0.001
Replacement procedure, n(%)	52 (42.3%)	450 (73.1%)	0.001
Post-operation fever, n(%)	18 (14.6%)	41 (6.7%)	0.003
ICD, n(%)	8 (6.5%)	20 (3.2%)	0.142

*ICD* implantable cardioverter defibrillator

Table 4 showed that, apart from male and replacement procedure, the followings were also statistically different between infection group and un-infection group, such as age, the use of antibiotics, post-operation fever and ICD (all, *P* < 0.05).

In bivariate analysis, gender was significantly associated with infection (*P* = 0.006). In multivariate logistic regression analysis controlling for postoperative fever at revision, gender (OR = 4.176, 95% CI: 1.647–10.590), age (OR = 1.041, 95% CI: 1.015–1.067) and replacement procedure (OR = 0.082, 95% CI: 0.038–0.178) remained significant predictors of infection (*P* < 0.01). But antibiotics lost the predicted effects (*P* > 0.05) (Table 5). As shown in Fig. 2, a ROC curve analysis demonstrated high sensitivity and specificity of replacement procedure, gender, and age for the identification of CIEDI. Further, based on 1000 samples, bootstrap analyses results were robust and fitting for the previous analysis.

**Table 5:** Multivariable logistic regression analysis after re-sampling (*n* = 739)

Variables	Β	SE	OR	95%CI	*P* value
Age	0.040	0.013	1.041	1.015–1.067	0.002
gender	1.429	0.475	4.176	1.647–10.590	0.003
replacement procedure	−2.498	0.394	0.082	0.038–0.178	0.000
antibiotics	−23.263	13,197	0.000	0.000–0.000	0.999

*CI* confidence intervals, *SE* standard error

### Accuracy rate results

After removing the incomplete data, the number of patients in original dataset is 4607 (4579 + 28). By SMOTE algorithm, the number of CIEDI patients was synthesized from 28 cases to 3047 (re-sampling ratio = 1088%), thus obtaining a dataset of 7626 (4579 + 3047) cases. Simultaneously, we expanded the original dataset proportionally (165%) to 7626 samples. The three different classification models of ANN, RF, and SVM were used for two rounds of experiments.

The results revealed that RF was the best classifiers by achieving accuracy rates (F_1_ score) of 22.3 and 93.1% in both original and re-sampling phases, respectively. There was a 70.8% improvement in accuracy rate. ANN and SVM had similar performance, by achieving accuracy rates of 17.9 and 93.4%; 18.9 and 92.7%; respectively. There was a 75.5 and 73.8% improvement in accuracy rate, respectively.

### Symptoms and signs/ microbiology

Of the 28 infections cases, the symptoms and signs identified were discharge/drainage = 16 (57.1%); impending erosion = 16 (57.1%); redness /discoloration = 16 (57.1%); swelling = 15 (53.6%); abscess/purulent liquid = 12 (42.9%); fever/chills = 10 (35.7%); local pain = 7 (25.0%); warmth = 1 (3.6%). Two of 28 patients were diagnosed with infectious endocarditis according to Duke’s criteria.

Of the 28 infections cases, cultures (pocket or lead or blood) were done in 25 (89.3%) of all infections, the remaining 3 patients (10.7%) were not cultured due to various reasons. Of these 25 patients with culture results, organisms primarily responsible were coagulase negative staphylococcus in 5 cases (20%) [Three grew staphylococcus epidemidis, one grew staphylococcus lentos, and one grew staphylococcus capitis]. *Staphylococcus aureus* grew in 8 cases (32%). Two cases (8%) grew mixed flora (*Staphylococcus aureus* and acinetobacter baumannii, staphylococcus epidermis and staphylococcus lentus), and organisms did not grow in 10 cases.

## Discussion

CIEDI remains a troublesome clinical problem, since CIEDI not only results in prolonged hospitalization and increased healthcare costs, but also relates to worse outcome and higher mortality [20]. Studies have been initiated to evaluate the risks of CIEDI and subsequently to improve and develop more effective and targeted strategies to prevent the CIEDI [21]. But in biomedical data, the imbalanced data problem occurs frequently [2]. Our data showed a high imbalance in the sizes of the patients with CIEDI profiles. The imbalanced data causes poor prediction performance for minority classes, because the algorithms is based on the assumption that the number of examples of classes is almost equal [2]. So, we should pay attention to the imbalanced data and select the appropriate analytical methods.

### Methods for addressing imbalance

There are usually two ways to handle the class imbalance problem and broadly distinguish the data into “classification level” or “data level”. The classification level, i.e. algorithm level, or the internal approaches refers the method to create new algorithms or modify existing ones to diminish the class imbalance problem [22]. This approach is reasonable in the case of datasets with only few classes, which have an equal probability to appear in practical scenarios. However, with the increasing number of classes in the collected object datasets, it is becoming impractical to provide equal representations for all classes in the training and testing subsets [22].

The data level or the external approach directly uses existing algorithms, but resample the data presented to these algorithms to diminish the effect of class imbalance [10]. Re-sampling method serves as a major tool of the data level approach [9]. Oversampling does not generate any new information, even lead to over-fitting, while under-sampling may remove important examples, even cause the classifier to miss important concepts [5]. To avoid this drawback, it is necessary to take advantage of the SMOTE algorithm [9]. SMOTE algorithm incorporates synthetic minority samples, based on the similarity between them, considering its K-nearest neighbors [5].

In this work, the data in each cluster was complete and the new synthetic samples were generated. Then, these new samples were used in our cluster-based approach to offset the missing observations. In the end, the analysis results of newly generated samples were robust and fitting for the primary analysis. Therefore, a re-sampling methodology can balance the data.

### Risk factors of early CIEDI

CIEDI has attracted more attention and many studies have investigated the risk factors [23]. Among the well-known reported risks, such as diabetes mellitus, end-stage renal disease, corticosteroid use, and so on [24], all procedure-related factors contributed to the early CIEDI while some comorbidities were associated with late infection [23]. Klug et al. reported that CIEDI occurred in 0.68% of patients within the first year after the device implantation or replacement [25]. Our data was similar and suggested that 60.7% (17/28) of infected patients occurred in the first year. Staphylococcal species were predominant in the present investigation, similar to the most published series [26].

Many centers now utilize peri-operative antibiotics as a preventative approach to reduce the occurrence of CIEDI [27]. This approach has been supported by previously studies [15, 28]. Nevertheless, there is still no consensus between American and European guidelines regarding the use of peri-operative antibiotics as a Class I indication [28, 29].

In this study, we found that there was no difference in CIEDI between no-antibiotics and peri-operative antibiotics, which is different from the previous study [28]. Occurrence of device infection in our series was roughly equal between pacemaker and ICD, which was not yet keeping with previous reports [30].

However, we also found a higher risk of infection with replacement compared with primary implantation. This was keeping with previous reports [31]. Above all, female had a lower device-related early infection rate compared to male.

### Evaluation

A measure that addresses accuracy issues is area under ROC which is a plot of the false positive rate to the true positive rate for all possible prediction thresholds [32]. ROC has also been used to compare performance of classifiers trained on imbalanced datasets [32]. In this study, as shown in Fig. 2, whether before or after re-sampling, replacement procedure had the larger AUC. This suggests a potential role of the replacement procedure in prediction of patients at risk for CIEDI.

Based on SMOTE algorithm, re-sampling is an effective method to deal with relatively small imbalanced datasets. But whether the accuracy rate is affected or not is our concern. The results showed us that regardless of the difference in performance of different classifiers, the re-sampling has a significant improvement on the accuracy of the classification models. This suggests that re-sampling could improve the accuracy of classification.

### Study limitations

First, a large enough data set was not given. Second, there were many factors that influence pacemaker infection. We only review five of them. Third, as the procedures were carried out in two major hospitals, details could not be obtained. Fourth, some complications may have been underestimated due to their severity not obtained in this database. Fifth, re-sampling technique can achieve class balance, but also potentially hinder the learning task. Above all, re-sampling technique didn’t provide anything new. Hybrid of methods is a favorable approach that combines multiple techniques from one or both above mentioned categories [32]. In the next step, we will further enrich the number and content of the dataset and study the impact of different re-sampling methods on machine learning performance.

## Conclusions

The application of re-sampling techniques can generate useful synthetic samples for the classification of imbalanced data and improve the accuracy of predicting efficacy of CIEDI. The peri-operative assessment should be intensified in male and aged patients as well as patients receiving replacement procedures for the risk of CIEDI.

## Data Availability

Data are available from the corresponding author on reasonable request due to privacy or other restrictions.

## Abbreviations

CI: Confidence intervals
CIED: Cardiac implantable electronic devices
CIEDI: Cardiac implantable electronic devices infection
CRT: Cardiac resynchronization therapy
ICD: Implantable cardioverter-defibrillator
SE: Standard error
